# Supplementary material for: Effect of Chemical Chaperones on the Stability of Proteins during Heat– or Freeze–Thaw Stress
Source: Int J Mol Sci. 2023 Jun 18;24(12):10298. doi: 10.3390/ijms241210298 (PMC10299496; doi:10.3390/ijms241210298)
Supplement: Supplementary file 1 [file ijms-24-10298-s001.zip › ijms-2439692-supplementary.pdf]

International Journal of *Molecular Sciences*

Supplementary Materials

Effect of Chemical Chaperones on the Stability of Proteins during Heat– or Freeze–Thaw Stress

Vera A. Borzova, Tatiana B. Eronina, Valeriya V. Mikhaylova, Svetlana G. Roman, Andrey M. Chernikov and Natalia A. Chebotareva *

Bach Institute of Biochemistry, Federal Research Centre "Fundamentals of Biotechnology" of the Russian Academy of Sciences, Leninsky pr. 33, 119071 Moscow, Russia; vera.a.borzova@gmail.com (V.A.B.); eronina@inbi.ras.ru (T.B.E.); mikhaylova.inbi@inbox.ru (V.V.M.); svetabaj@gmail.com (S.G.R.); chernikov.andrei.m@gmail.com (A.M.C.)

* Correspondence: n.a.chebotareva@gmail.com

S1. The estimation of the aggregated GDH fraction by AUC

Table S1. The fraction of aggregated GDH (γ_{agg}) estimated from the amount of the protein precipitated during centrifugation after the freeze-thaw-induced denaturation.

GDH + chemical chaperone	Chaperone concentration, mM	γ_{agg} (%)
No additives		95
+ HPCD	+ 1 µM HPCD	90
	+ 10 µM HPCD	92
	+ 100 µM HPCD	87
	+ 1 mM HPCD	92
	+ 50 mM HPCD	32
	+ 75 mM HPCD	0
	+ 100 mM HPCD	0
	+ 150 mM HPCD	0
	+ 175 mM HPCD	0
+ Tre	+ 50 µM Tre	84
	+ 1 mM Tre	86
	+ 10 mM Tre	88
	+ 25 mM Tre	68
	+ 50 mM Tre	57
	+ 75 mM Tre	8
	+ 100 mM Tre	5
	+ 150 mM Tre	3
	+ 200 mM Tre	0
	+ 350 mM Tre	0
+ Sorb	+ 50 mM Sorb	0
	+ 100 mM Sorb	0

S2. Refractive indices, density and dynamic viscosity of chemical chaperones solutions

Table S2. The values of refractive index (*n*), density (ϱ) and dynamic viscosity (η) of the chemical chaperones solutions at 25 °C and 37 °C

Concentration, mM	*n*	ϱ, g/cm³	η, mPa·s
**HPCD**			
25 °C			
0 (0.1 M Na-phosphate buffer, pH 7.6, 10 mM NaCl)	1.33489 ± 0.00002	1.0101 ± 0.0005	0.9442 ± 0.0001
50	1.34300 ± 0.00002	1.0281 ± 0.0005	1.1346 ± 0.0004
75	1.34572 ± 0.00002	1.0348 ± 0.0005	1.2264 ± 0.0004
100	1.35089 ± 0.00002	1.0462 ± 0.0005	1.4048 ± 0.0002
120	1.35261 ± 0.00002	1.0498 ± 0.0005	1.4660 ± 0.0003
150	1.35709 ± 0.00002	1.0588 ± 0.0005	1.6866 ± 0.0003
175	1.36012 ± 0.00002	1.0673 ± 0.0005	1.8785 ± 0.0004
0 (30 mM Hepes, pH 6.8, 150 mM NaCl)	1.33698 ± 0.00002	1.0067 ± 0.0005	0.9238 ± 0.0005
75	1.34830 ± 0.00002	1.0361 ± 0.0005	1.2614 ± 0.0005
37 °C			
30 mM Hepes, pH 6.8, 150 mM NaCl	1.33480 ± 0.00002	1.0040 ± 0.0005	0.7267 ± 0.0001
75	1.34610 ± 0.00002	1.0302 ± 0.0005	0.9325 ± 0.0005
**Tre**			
25 °C			
0 (0.1 M Na-phosphate buffer, pH 7.6, 10 mM NaCl)	1.33489 ± 0.00002	1.0101 ± 0.0005	0.9442 ± 0.0001
50	1.33716 ± 0.00002	1.0161 ± 0.0005	0.9830 ± 0.0010
75	1.33832 ± 0.00002	1.0190 ± 0.0005	1.0160 ± 0.0040
100	1.33944 ± 0.00002	1.0222 ± 0.0005	1.0290 ± 0.0020
150	1.34171 ± 0.00002	1.0278 ± 0.0005	1.0729 ± 0.0008
200	1.34388 ± 0.00002	1.0338 ± 0.0005	1.1260 ± 0.0002
350	1.35211 ± 0.00002	1.0553 ± 0.0005	1.3433 ± 0.0008
500	1.35683 ± 0.00002	1.0067 ± 0.0005	1.5010 ± 0.0070
37 °C			
30 mM Hepes, pH 6.8, 150 mM NaCl	1.33480 ± 0.00002	1.0040 ± 0.0005	0.7267 ± 0.0001
350	1.35050 ± 0.00002	1.0457 ± 0.0005	1.0000 ± 0.0050
**Sorb**			
25 °C			
0 (0.1 M Na-phosphate buffer, pH 7.6, 10 mM NaCl)	1.33478 ± 0.00002	1.0094 ± 0.0005	0.9430 ± 0.0010
10	1.33506 ± 0.00002	1.0102 ± 0.0005	0.9480 ± 0.0010
20	1.33533 ± 0.00002	1.0107 ± 0.0005	0.9490 ± 0.0010
30	1.33554 ± 0.00002	1.0112 ± 0.0005	0.9528 ± 0.0004
50	1.33606 ± 0.00002	1.0125 ± 0.0005	0.9580 ± 0.0010
100	1.33738 ± 0.00002	1.0158 ± 0.0005	0.9858 ± 0.0002

300	1.34233 ± 0.00002	1.0277 ± 0.0005	1.0890 ± 0.0030
500	1.34749 ± 0.00002	1.0401 ± 0.0005	1.2063 ± 0.0001
700	1.35261 ± 0.00002	1.0520 ± 0.0005	1.3215 ± 0.0001
1000	1.36011 ± 0.00002	1.0702 ± 0.0005	1.5647 ± 0.0003
**Bet**			
25 °C			
0 (0.1 M Na-phosphate buffer, pH 7.6, 10 mM NaCl)	1.33504 ± 0.00002	1.0105 ± 0.0005	0.9268 ± 0.0001
1	1.33501 ± 0.00002	1.0103 ± 0.0005	0.9270 ± 0.0001
10	1.33502 ± 0.00002	1.0105 ± 0.0005	0.9284 ± 0.0004
25	1.33527 ± 0.00002	1.0109 ± 0.0005	0.9307 ± 0.0002
50	1.33560 ± 0.00002	1.0112 ± 0.0005	0.9341 ± 0.0001
100	1.33646 ± 0.00002	1.0120 ± 0.0005	0.9382 ± 0.0005
1000	1.34894 ± 0.00002	1.0204 ± 0.0005	1.1420 ± 0.0010
37 °C			
30 mM Hepes, pH 6.8, 150 mM NaCl	1.33480 ± 0.00002	1.0040 ± 0.0005	0.7267 ± 0.0001
500	1.34150 ± 0.00002	1.0113 ± 0.0005	1.8051 ± 0.0010
